# Fluid and hemodynamic management in hemodialysis patients: challenges and opportunities

**DOI:** 10.1590/2175-8239-JBN-2019-0135

**Published:** 2019-10-24

**Authors:** Bernard Canaud, Charles Chazot, Jeroen Koomans, Allan Collins

**Affiliations:** 1 Montpellier University, Montpellier, France.; 2 Senior Medical Scientist, Global Medical Office, FMC Deutschland, Bad Homburg, Germany.; 3 Head of Clinical Governance, NephroCare France, Fresnes, France.; 4 Maastricht University Medical Center, Department of Internal Medicine, Division of Nephrology, Netherlands.; 5 University of Minnesota, Minneapolis Minnesota, USA.; 6 Senior Medical Scientist, Global Medical Office, FMC North America, Waltham, MA, USA.

**Keywords:** Water-Electrolyte Balance, Hemodynamic Monitoring, Blood Pressure, Cardiovascular Deconditioning, Renal Dialysis, Treatment Outcome, Manejo Hídrico e de Sódio, Monitorização Hemodinâmica, Pressão Sanguínea, Descondicionamento Cardiovascular, Hemodiálise, Resultado do Tratamento

## Abstract

Fluid volume and hemodynamic management in hemodialysis patients is an essential component of dialysis adequacy. Restoring salt and water homeostasis in hemodialysis patients has been a permanent quest by nephrologists summarized by the ‘dry weight’ probing approach. Although this clinical approach has been associated with benefits on cardiovascular outcome, it is now challenged by recent studies showing that intensity or aggressiveness to remove fluid during intermittent dialysis is associated with cardiovascular stress and potential organ damage. A more precise approach is required to improve cardiovascular outcome in this high-risk population. Fluid status assessment and monitoring rely on four components: clinical assessment, non-invasive instrumental tools (e.g., US, bioimpedance, blood volume monitoring), cardiac biomarkers (e.g. natriuretic peptides), and algorithm and sodium modeling to estimate mass transfer. Optimal management of fluid and sodium imbalance in dialysis patients consist in adjusting salt and fluid removal by dialysis (ultrafiltration, dialysate sodium) and by restricting salt intake and fluid gain between dialysis sessions. Modern technology using biosensors and feedback control tools embarked on dialysis machine, with sophisticated analytics will provide direct handling of sodium and water in a more precise and personalized way. It is envisaged in the near future that these tools will support physician decision making with high potential of improving cardiovascular outcome.

## Resumo

O volume de fluidos e o controle hemodinâmico em pacientes em hemodiálise é um componente essencial da adequação da diálise. A restauração da homeostase do sal e da água em pacientes em hemodiálise tem sido uma busca constante por parte dos nefrologistas, no que condiz à abordagem do “peso seco. Embora essa abordagem clínica tenha sido associada a benefícios no desfecho cardiovascular, recentemente tem sido questionada por estudos que mostram que a intensidade ou agressividade para remover fluidos durante a diálise intermitente está associada a estresse cardiovascular e dano potencial a órgãos.para remover fluidos durante a diálise intermitente está associada a estresse cardiovascular e dano potencial a órgãos. Uma abordagem mais precisa é necessária para melhorar o desfecho cardiovascular nessa população de alto risco. A avaliação e monitorização do estado hídrico baseiam-se em quatro componentes: avaliação clínica, ferramentas instrumentais não invasivas (por exemplo, US, bioimpedância, monitorização do volume sanguíneo), biomarcadores cardíacos (e.g. peptídeos natriuréticos), algoritmos e modelagem de sódio para estimar a transferência de massa. O manejo otimizado do desequilíbrio hídrico e de sódio em pacientes dialíticos consiste em ajustar a remoção de sal e líquido por diálise (ultrafiltração, dialisato de sódio), e restringir a ingestão de sal e o ganho de líquido entre as sessões de diálise. Tecnologia moderna que utiliza biosensores e ferramentas de controle de feedback, hoje parte da máquina de diálise, com análises sofisticadas, proporcionam o manejo direto sobre o sódio e a água de uma maneira mais precisa e personalizada. Prevê-se no futuro próximo que essas ferramentas poderão auxiliar na tomada de decisão do médico, com alto potencial para melhorar o resultado cardiovascular.

## Fluid and hemodynamic management in hemodialysis patients: An identified modifiable cardiovascular risk factor

Optimal fluid volume management in dialysis patients is an essential component of dialysis adequacy but amplitude of volume fluctuation is still a quite challenging clinical condition^1^. Restoring salt and water homeostasis in hemodialysis patients has been a permanent Holy Grail quest by nephrologists from the sixties. Salt and water management in dialysis patients is frequently summarized by the ‘dry weight’ approach^3^^,^ 4. Although this clinical approach has been associated with benefits on cardiovascular outcome, it is now challenged by recent studies showing that intensity or aggressiveness to remove fluid during conventional thrice-weekly dialysis might induce excessive hemodynamic stress and potential organ damage with potentially deleterious consequences on the long term^5^^,^ 6. In brief, ‘dry weight’ policy is necessary from a clinical perspective but it is not sufficient from a pathophysiologic perspective to ensure a fully cardioprotective effect in dialysis patients. A more balanced and precise approach is required to improve cardiovascular outcome in this high-risk population. To satisfy this unmet need, it is time to move to a broader approach embracing the whole hemodynamic management of dialysis patients rather than focusing only on their fluid management^7^.

**Figure 1:**
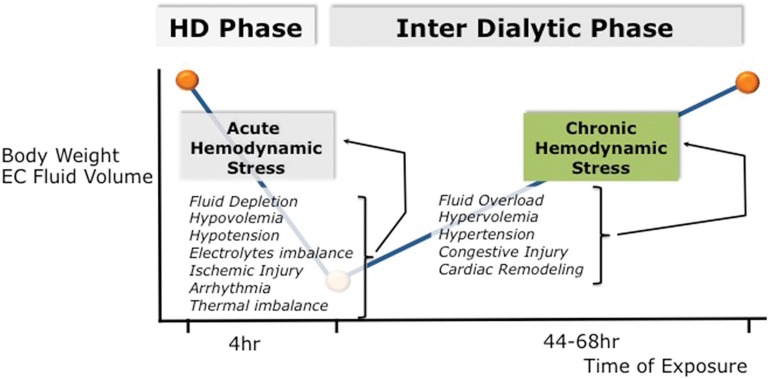
Hemodynamic Stress in HD Patient : Acute vs. Chronic Cardiovascular Stress.

Intermittent renal replacement therapy exposes dialysis patients to continuous and repetitive hemodynamic stress conditions (Figure 1). By nature this is due to intermittency of treatment exposing patients to up (interdialytic period) and down (intradialytic period) fluid volume changes. This is best summarized by the “unphysiological profile” of short intermittent dialysis treatment^8^^,^ 9. From a mechanistic approach, one can identify two different stress conditions: firstly, a chronic hemodynamic stress phase, which reflects extracellular fluid accumulation, often superimposed on a status of chronic fluid expansion during the interdialytic period; secondly, an acute hemodynamic stress phase, which reflects intravascular fluid depletion induced by dialysis session (ultrafiltration and sodium removal) marked by critical hypovolemia leading eventually to hypotensive episodes and impaired organ perfusion^10^.

**Figure 2:**
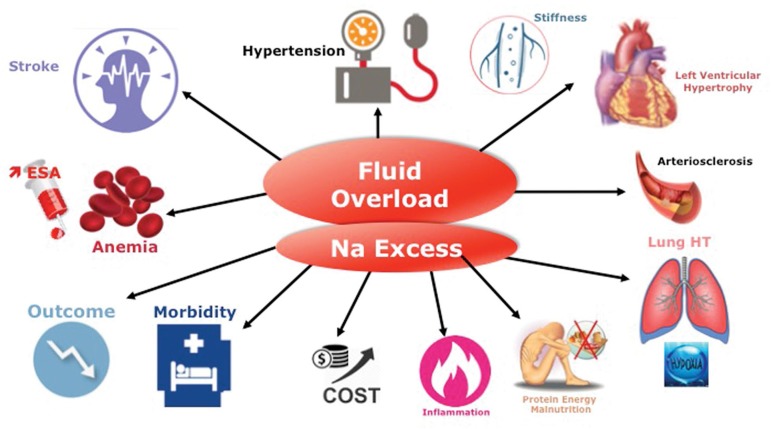
Chronic Hemodynamic Stress: Chronic Fluid Overload and Its Consequences.

**Figure 3:**
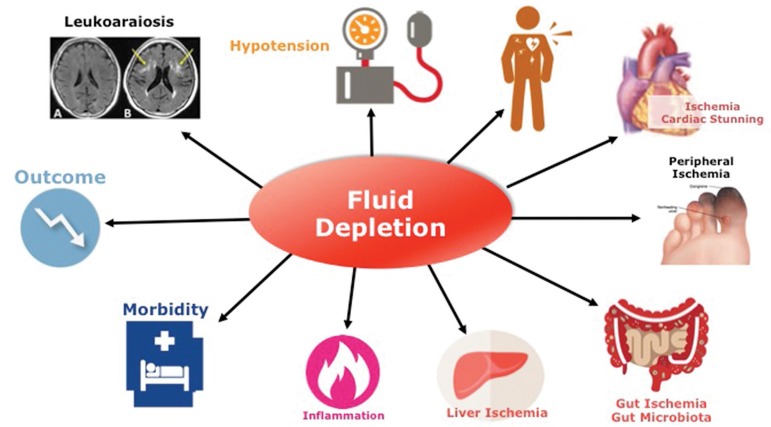
Acute Hemodynamic Stress : Excessive or Aggressive Fluid Depletion.

Sodium and fluid accumulation that may occur in dialysis patients over time due to repetitive positive fluid imbalance is responsible for chronic extracellular fluid overload (Figure 2) with its adverse effects and cardiovascular consequences leading to poor outcomes^11^. Extracellular fluid overload and poor fluid management are the basic fundament of cardiovascular complications in hemodialysis patients^12^^,^ 13. Hypertension as part of this constellation of disorders is widely recognized as a leading cause for left ventricular cardiomyopathy and accelerated atherosclerosis including coronary artery disease, peripheral artery disease, and cerebrovascular disease^14^^,^ 15 ^,^ 16. Interestingly, as shown in a recent large cohort study, the presence of fluid overload per se has an independent and additive deleterious effect on blood pressure (either low or high blood pressure) in dialysis patient outcomes, which increases the global negative impact of blood pressure per se^17^. Hyponatremia, for reasons not entirely understood, is also associated with poor outcome in dialysis patients^18^^,^ 19 ^,^ 20. Management of sodium and fluid excess to restore fluid status homeostasis, (Figure 3) either by moderate or high ultrafiltration rate, or high plasma-to-dialysate sodium concentration gradient leading potentially to critical hypovolemia, is also associated with increased risk of mortality^21^^,^ 22 ^,^ 23 ^,^ 24 ^,^ 25 ^,^ 26. Combination of these characteristics increases significantly the negative impact of each one on patient outcome^27^^,^ 28 ^,^ 29 ^,^ 30. In this context, salt and fluid management of dialysis patients represents a major challenge for clinicians.

## Fluid and hemodynamic management in hemodialysis patients: Challenges

**Figure 4:**
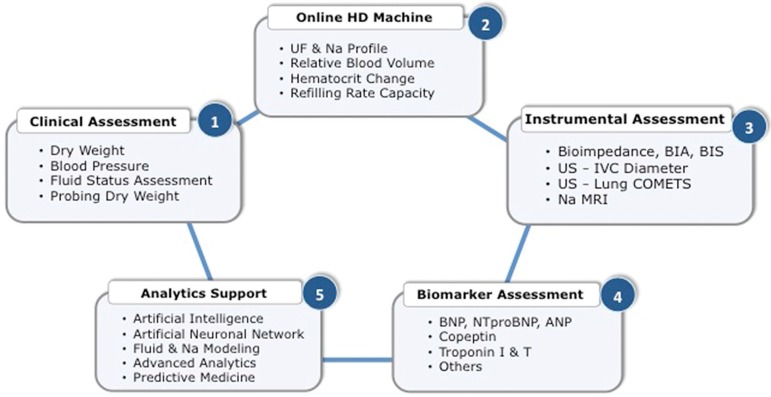
Fluid and Hemodynamic Monitoring in HD Patients - Usual Workflow.

Assessing fluid status of dialysis patients is not an easy task from a clinical perspective. In that context, it is interesting to note that over time several tools have been proposed (Figure 4) to assess salt and water status in hemodialysis patients with a common objective of monitoring and guiding caregivers in their prescription^31^^,^ 32.

1. Clinical assessment focusing on fluid status, hemodynamic stability, and patient perception was the first attempt to address this issue in developing the concept of ‘dry weight’^33^^,^ 34. It relates in fact to the post-dialysis weight at which dialysis the patient has - in theory - no sign of fluid imbalance (neither excess nor depletion), blood pressure values in normal range for his or her clinical condition, and feels comfortable without functional limitations^35^. ‘Dry weight’ is probed over time by clinicians and reassessed periodically according to the patient conditions, changes, and/or intercurrent events in order to keep its fluid status balance over time^36^. Further work has led investigators to refine assessment of the ‘dry weight’ concept^37^.

Subsequently, several tools have been proposed to help physicians in refining clinical acumen and defining more objectively ‘dry weight’ of dialysis patients^38^^,^ 39. In brief, they utilize either instrumental tools or biomarkers^40^^,^ 41 ^,^ 42.

2. Instrumental or technology-based tools use various non-invasive ways to assess volemia, fluid status, or hemodynamic surrogate indicators.

Inferior vena cava diameter (IVCD) and collapsibility has been proposed to monitor intravascular volume and right atrial pressure or central venous pressure in dialysis patients with interesting findings^43^^,^ 44 ^,^ 45. However, the practical difficulty in implementing these methods in a dialysis facility and the poor predictive value on blood pressure response in probing dry weight have precluded its generalizability^46^. However, recent data in critically ill patients showed that IVCD collapsibility had reasonable value (c-statistic 0.72) in predicting tolerance to fluid removal^47^.

Relative blood volume change (RBV) and refilling rate capacity during dialysis assessed by online blood volume sensor has been also proposed for fluid management. In expert hands, this tool provides useful information on individual patient volume status to facilitate hemodynamic guidance^49^. Furthermore, blood volume monitoring has been shown helpful to set individual patient critical volemia beyond which occurrence of severe intradialytic hypotension is likely to occur^50^. Despite the fact that most studies using blood volume monitoring (BVM) devices are reporting positive feedback on blood pressure control and hemodynamic stability^51^, their clinical benefit is still matter of controversy^52^. Furthermore, in a randomized controlled trial (CLIMB Study) comparing BVM guided treatment versus standard of care had negative results that were associated with adverse outcomes^53^. In a more recent study, BVM guided feedback did not result in an improvement in intradialytic hemodynamic stability although also no evidence of harm was found^54^. Absolute blood volume measurement, based on non-invasive measurement either by dilution or online calculation, has been proposed recently for a better assessment of this crucial parameter^55^^,^ 56 ^,^ 57. To date, no study has explored the clinical benefits of monitoring precisely this parameter.

Bioimpedance approach has been proposed over the last few years as a more objective way to assess fluid status in dialysis patients^58^^,^ 59. Several approaches (segmental versus total body, single versus multifrequency) using various devices and algorithms have been developed with interesting results^60^. In a systematic review, multifrequency bioimpedance spectroscopy (BIS) analysis [NICE, UK; CADTH, Canada] was recognized as the most precise and reliable tool in a clinical setting for guiding fluid management in dialysis patients at present available, although its use has not entered clinical guidelines yet^61^^,^ 62. In addition, extensive use of BIS in clinical studies has generated substantial evidences showing that BIS was able to detect subtle fluid volume variation^63^^,^ 64 ^,^ 65 and to support the notion that more precise fluid management might improve intermediate outcomes and dialysis patient endpoints^66^^,^ 67 ^,^ 68. Few prospective clinical trials in advanced kidney disease or dialysis patients are ongoing to define more precisely the value of BIS in managing fluid status and its impact on preservation of residual kidney function and on cardiovascular outcomes^69^^,^ 70.

More recently, it has also been proposed to extend the use of lung ultrasound in chronic hemodialysis patients for tracking silent fluid accumulation in the lung interstitium (extravascular edema). Interlobular septa thickening due to water accumulation reflects US beam and generates visible B line bundles (comet-like tail). A simple counting of these B lines provides an estimate of lung water excess and predictive value for patient outcomes^71^^,^ 72.

Sodium MRI has been introduced quite recently in the field of sodium and fluid assessment in chronic kidney disease patients in dialysis to assess tissue sodium accumulation^73^. Tissue sodium, namely ‘free-water sodium’ or ‘sodium bound to proteoglycans’, accumulates in chronic kidney disease and modulates lymphangiogenesis and blood pressure via proinflammatory resident cells^74^^,^ 75. Recent studies have shown that tissue sodium might contribute to systemic toxicity via local tissue and organ damage^76^^,^ 77. Left ventricular hypertrophy is positively associated with the amount of tissue sodium storage independent from blood pressure^78^. Vascular stiffness is also associated with sodium intake and sodium tissue storage independent from mechanical stress^79^^,^ 80. Furthermore, sodium tissue accumulation might contribute to metabolic and inflammatory disorders (e.g., insulin resistance, protein energy wasting) that increase cardiovascular risk. Due to its complex setting and limited number of scanning devices, sodium MRI remains an experimental tool with quite restricted access. However, it is envisioned that in the near future the dedicated extremity sodium MRI device, such as in rheumatologic field, could be used in a more systematic way to assess salt tissue content in dialysis patients^81^.

3. Cardiac and vascular biomarkers have been used extensively in an attempt to disentangle fluid status and cardiac dysfunction in dialysis patients. Atrial natriuretic peptides (ANP, BNP, and NT-proBNP) are the most popular ones for assessing fluid overload^82^^,^ 83 while, on the other hand, copeptin (a vasopressin precursor) is more reflective of fluid depletion^84^. Cardiovascular biomarkers reflecting cardiac or endothelium injury are also of interest to set a more precise and personalized fluid management approach. Sensitive troponin family markers (troponin I and T) have been used to detect critical myocardial hypoperfusion. In this context, troponins (I and/or T) reflecting cardiac injury appear to be the most appealing ones being correlated with hemodynamic stress intensity, bearing a high predictive value for future cardiac events^85^^,^ 86 ^,^ 87 ^,^ 88. Several other cardiac and endothelial biomarkers (e.g., ADMA, FG23, ROS, NO pathways) appear promising either isolated or combined in assessing cardiovascular risk but deserve further studies to define their exact place in fluid management strategy since they reflect tissue remodeling, uremic byproducts, inflammation, or oxidative stress mechanisms^89^^,^ 90 ^,^ 91. Although these cardiac biomarkers have been shown to be quite useful for patient risk stratification, their predictive (specificity and sensitivity) and/or clinical value in term of fluid status management has remained limited to clinical cases management^92^. Interpretation and clinical application of these cardiac biomarkers should be done with caution and be integrated in strategic care planning of dialysis patients since their circulating levels reflect not only fluid status but also kidney function deterioration and cardiac remodeling^93^^,^ 94. Multi-markers approach and time trend analysis of these biomarkers have been proposed to better support physician decision in stratifying cardiovascular risk but raising a cost issue^95^^,^ 96.

4. In recent past years, several researchers have develop algorithms to quantify sodium and water mass transfer during hemodialysis sessions using either mass balance equations based on the law of conservation of mass within the dialysis/patient system^97^^,^ 98 or by modeling sodium mass transfer using ionic dialysance with dialysate and plasma sodium concentrations^99^. Interestingly, on one hand, these studies have confirmed the validity of such an approach by direct dialysis quantification using partial or total dialysate collection. On the other hand, it has been clearly shown that sodium and water mass transfer and kinetics might be considered as a patient profile characteristic; in other words, intra-individual variability was relatively narrow while inter-individual variability was tremendously high. Several putative causal factors are associated with individual sodium and water profile (sodium or osmotic set-point) but most likely reflecting life style and diet observance^101^^,^ 102 ^,^ 103. In addition, it has been shown that acting on sodium and water mass transfer by individualizing dialysis sodium prescription it was possible to alter patient perception (reduce thirst, and sodium and water intake), reduce interdialytic weight gain, and modify outcomes (reduce dry weight and arterial blood pressure)^104^^,^ 105 ^,^ 106. However, this approach is hampered by the need for frequent lab sampling, which is cumbersome for clinical practice.

## Fluid and hemodynamic management in hemodialysis patients: Opportunities

Optimal management of fluid and sodium imbalance in dialysis patients is achieved by adjusting salt and fluid removal through dialysis and salt intake restriction, and fluid gain between dialysis sessions. This is the conventional approach obtained by adjusting ‘dry weight’ according to clinical judgment and complementary tools including dialysate sodium prescription adaptation described earlier. However, this approach may be hampered by the discontinuous nature of the HD treatment and/or patient intolerance to fluid and sodium removal. An obvious solution would be to increase time and/or frequency of dialysis sessions in patients with high inter-dialytic weight gains and/or intolerance for fluid removal, as this has been shown to reduce intradialytic hemodynamic stress. However, this approach will not always be possible for financial or logistic reasons, or because of the wish of the patient.

Modern technology using biosensors and sophisticated analytics provide tools for handling directly sodium and water during hemodialysis session in a more precise and personalized way that have potential for improving patient outcome^109^. In this context, the use of calibrated conductivity meters or microsensors placed on dialysis fluid paths both inlet and outlet have been used to develop specific algorithms able to determine the precise contribution of sodium salt among the bulk of electrolytes^110^^,^ 111. Furthermore, the disposition of sensors on the dialysate path offers a means to ensure a precise mass balance due to a closed circuit. In addition, the combined use of advanced analytics embedded in the central processor unit provides a way to ensure direct handling of sodium and water according to the targeted prescription and patient baselines. Artificial intelligence has been recently proposed for clinical guidance and decision-making support in adapting dialysis prescription (e.g., ultrafiltration rate, dialysate sodium, treatment time) to ensure an optimal fluid status control and to minimize hemodynamic stress. The added value of these tools needs to be assessed in the future by clinical studies.

Complementary clinical studies on a large scale should help to better characterize dialysis patients in term of diet sodium intake over prolonged time period and explore effects of this precise sodium and fluid management approach on patients intermediary and clinical endpoint outcomes.

## Take home message

Dialysis adequacy concept has evolved over time and based on patient outcomes. Due to more efficient hemodialyzers, more technically advanced hemodialysis machines, and wider use of ultrapure dialysis fluid, efficiency and biocompatibility of renal replacement therapy have improved tremendously^114^^,^ 115 ^,^ 116. Cardioprotective hemodialysis requires further decisive actions in which sodium and fluid management are top ranking^117^. However, restoring homeostasis of extracellular volume, achieving adequately blood pressure control, and preserving hemodynamic equilibrium of dialysis patients still remains a matter of concern^118^^,^ 119. Restoring sodium and fluid mass balance of dialysis patients is moving from an over simplistic ‘dry weight’ approach to a more precise fluid management approach with support of new diagnostic and monitoring tools and will enter in a new era with availability of smart hemodialysis machines with direct dialysis sodium and water handling options and with the support of advanced technology and analytics.
